# Evidence Mapping: Healthy Eating and Physical Activity Practice Elements in Early Childhood Education and Care

**DOI:** 10.1002/hpja.70170

**Published:** 2026-03-12

**Authors:** Melanie Lum, Alice Grady, Luke Giles, Heidi Turon, Nicole Pearson, Ana Renda, Luke Wolfenden, Sze Lin Yoong

**Affiliations:** ^1^ Global Centre for Preventive Health and Nutrition, Institute for Health Transformation, School of Health and Social Development, Faculty of Health Deakin University Geelong Victoria Australia; ^2^ School of Medicine and Public Health, College of Health, Medicine and Wellbeing The University of Newcastle Callaghan New South Wales Australia; ^3^ Population Health, Hunter New England Local Health District Wallsend New South Wales Australia; ^4^ Hunter Medical Research Institute New Lambton Heights New South Wales Australia; ^5^ National Centre of Implementation Science University of Newcastle Callaghan New South Wales Australia; ^6^ Population Health, Sydney Local Health District Camperdown New South Wales Australia

## Abstract

**Background:**

Many health and government organisations have developed recommendations to promote the healthy eating and physical activity of children attending early childhood education and care (ECEC) settings. However, the evidence supporting these recommendations is not well described. An examination of the current evidence is needed to support decision‐makers to understand and prioritise practices for implementation.

**Aim:**

To utilise a novel systematic evidence‐mapping process which: (i) examines the evidence‐base underpinning ECEC‐based healthy eating and physical activity practice elements; and (ii) classifies practice elements according to the World Health Organization (WHO) Standards for Healthy Eating, Physical Activity, Sedentary Behaviour and Sleep in Early Childhood and Care Settings to examine alignment with current global guidelines.

**Methods:**

We undertook a two‐stage, five‐step systematic process, involving identifying existing evidence and conducting a secondary data analysis and synthesis of the evidence underpinning practice elements.

**Results:**

Sixteen healthy eating and 19 physical activity practice elements were assessed as likely beneficial. Most of these mapped to WHO Standard 2: Creating supportive environments. Seven practice elements were assessed as possibly beneficial, two as possibly not beneficial and none as not beneficial. There was insufficient evidence to assess 39 practice elements.

**Conclusions:**

This study provides insights into the evidence underpinning practice elements included in ECEC‐based guidelines, identifies evidence‐based practice elements not included in existing guidelines and highlights opportunities where evidence can be strengthened.

**So What?:**

The evidence underpinning guideline recommendations is variable or non‐existent. Evaluation around the implementation of guidelines within funded programs is needed.

## 1 Background

Modifiable risk factors, such as poor dietary intake and physical inactivity, are the leading causes of death and disability from non‐communicable diseases [1]. As these risk factors track from childhood into later life [2, 3], leading health organisations and countries have released preventive health strategies that support healthy eating and physical activity in young children [4, 5, 6]. Early childhood and education care (ECEC) services are one setting in which population dietary intake and physical activity levels of children can be addressed at a critical developmental stage. ECEC services are regulated facilities which care for children aged 0–6 years prior to attending compulsory schooling, including long day care, preschools, nurseries, kindergartens and family day care (also known as family child care homes). In many developed countries, ECEC services are the primary setting outside of home where young children spend a significant portion of their time [7].

Many high‐income countries and health organisations have published guidance outlining best‐practice recommendations to create ECEC environments supportive of child diet and physical activity [8]. Examples of recommendations include offering a variety of food awareness/education activities and providing opportunities for adult‐led, structured physical activity [8]. An assessment of the methodological quality of the guidelines using the Appraisal of Guidelines for Research & Evaluation II (AGREE II) tool [9] indicated a lack of reporting of how evidence was gathered or used to develop the recommendations [8].

Since the release of such ECEC‐specific guidelines, there has been growth in high‐quality empirical research evidence in this area [10, 11]. In addition, in 2021, the World Health Organization (WHO) published global standards for ECEC settings to support the implementation of the WHO guidelines on healthy eating, physical activity, sedentary behaviour and sleep in young children [12]. The examination of new evidence, in the context of WHO standards, is needed to understand and prioritise recommendations for implementation within the sector [12].

Therefore, this study sought to examine this evidence building on the common practice elements approach which has been used to identify important evidence‐based elements for early childhood development [13, 14]. This approach seeks to identify shared, active components (i.e., ‘practice elements’) across multiple evidence‐based interventions, rather than focusing on single branded programs. Examples of common practice elements in ECEC‐based healthy eating and physical activity interventions include educator training, developing and adopting policy and encouraging family involvement [8]. Such an approach is intended to promote flexibility for scaling by allowing selection of components most relevant to the local context and support the development of training and implementation strategies by focusing on a ‘set’ of universal components. Drawing on systematic review and randomised controlled trial (RCT) evidence, the aims of this study are to utilise a novel systematic evidence‐mapping process which: (i) examines the evidence‐base underpinning ECEC‐based healthy eating and physical activity practice elements; and (ii) classifies practice elements according to the WHO Standards for Healthy Eating, Physical Activity, Sedentary Behaviour and Sleep in Early Childhood and Care Settings to examine alignment with current global guidelines.

## 2 Methods

**FIGURE 1:**
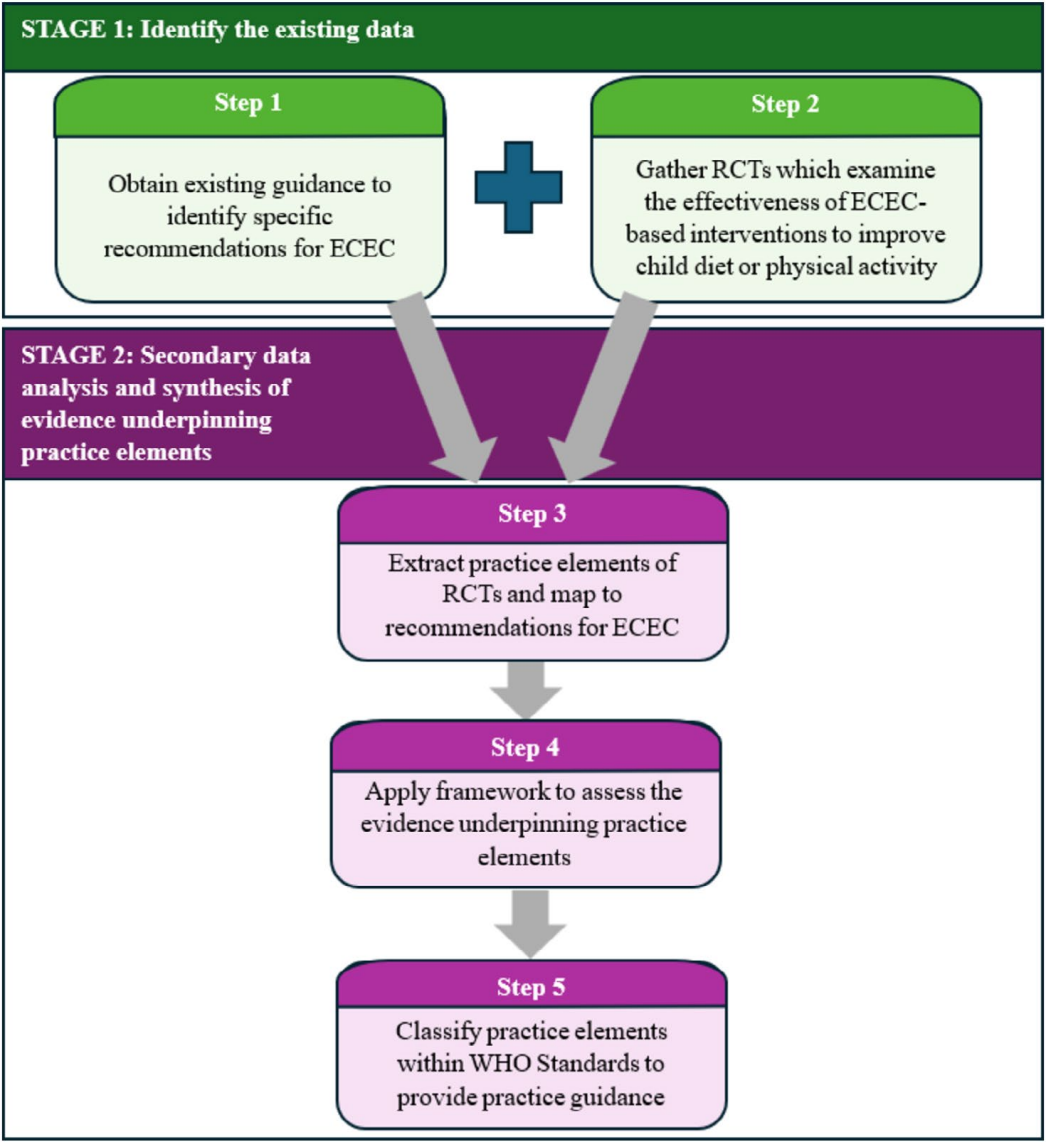
Two‐stage, five‐step systematic evidence‐mapping process to describe the evidence‐base underpinning practice elements of existing ECEC‐based healthy eating and physical activity guidelines.

This study employed a two‐stage, five‐step evidence‐mapping process using secondary data extracted from published RCTs included within high quality, contemporary systematic reviews (Figure 1). In accordance with the common practice elements approach [13, 14], we identified practice elements of existing ECEC‐based healthy eating and physical activity interventions using deductive, then inductive methods and then applied a framework to synthesise the evidence underpinning these practice elements.

### 2.1 Stage 1: Identify the Existing Evidence Base

#### 2.1.1 Step 1: Obtain Existing Guidance to Identify Specific Recommendations for ECEC

We obtained the findings of a previous systematic review of 38 ECEC‐based healthy eating and physical activity guidelines and policies conducted by the team [8], which included a list of broad practice themes encompassing specific recommendations. Briefly, policies and guidelines were included if they: (i) promoted obesity prevention policies or practice implementation within the ECEC setting; (ii) were aimed at improving obesity prevention behaviours of preschool children aged > 12 months and < 6 years old; (iii) were developed for contexts relevant to high‐income countries; and (iv) were published in English language. The search was conducted in September 2020.

#### 2.1.2 Step 2: Gather RCTs Which Examine the Effectiveness of ECEC‐Based Interventions to Improve Child Diet or Physical Activity

We gathered all RCTs captured within the following systematic reviews conducted by the team examining: (i) ECEC‐based healthy eating interventions [11]; (ii) ECEC‐based physical activity interventions (review of reviews) [10]; (iii) physical activity interventions delivered in centre‐based ECEC [15]; and (iv) healthy eating and physical activity interventions delivered in family day care settings [16]. A summary of the eligibility criteria for these reviews is provided in Table 1.

**TABLE 1:** A summary of the eligiblity criteria and date of search for the systematic reviews examined to gather RCTs of ECEC‐based interventions to improve child diet or physical activity.

Date of search	ECEC‐based healthy eating interventions [11]	ECEC‐based physical activity interventions (review of reviews [10])	Physical activity interventions delivered in centre‐based ECEC [15]	Healthy eating and physical activity interventions delivered in family day care settings [16]
	February 2022	November 2020	October 2022	March 2019
Participants	Children aged 6 months to 6 years (inclusive) attending ECEC services. Interventions that target parents, carers and ECEC staff as part of healthy eating interventions were also included.	Children aged 0–6 years attending ECEC.	Children aged 0 to 6 years attending ECEC services.	Children attending family day care, as well as their parents or carers, professionals responsible for the care provided to children whilst attending family day care.
Intervention	Healthy eating interventions delivered within an ECEC service *Exclusion*: Interventions that focused specifically on examining malnutrition or malnourishment, as well as those focusing on obesity‐management interventions (i.e., those that only included children classified as overweight or obese).	Intervention conducted in ECEC which target child physical activity.	Interventions conducted in centre‐based ECEC services with at least one component aimed at increasing physical activity among children. *Exclusion*: Interventions that focused on obesity‐management interventions (i.e., those that only included children classified as overweight or obese).	All interventions that promote healthy eating, feeding behaviour, nutrition, diet and/or physical activity, reduce sedentary behaviour or prevent unhealthy weight gain of children attending family day care services. *Exclusion*: Interventions that focused specifically on examining malnutrition or malnourishment were excluded, as were those examining obesity treatment (i.e., those included only overweight/obese children).
Comparator	Alternative intervention or usual care control.	With or without a control.	Alternative intervention or usual care control.	Alternative intervention or usual care control.
Outcomes	At least one dietary or physical (i.e., anthropometric) outcome that was assessed at least 3 months or longer from baseline.	Any quantitative measure of child physical activity via objective or validated methods.	Physical activity measured via accelerometer or pedometer.	Any objective or subjective measures of dietary intake, physical activity or sedentary behaviour and measures of weight status.
Study design	RCT	Systematic review	RCT	RCT, cluster‐RCT, factorial trial, interrupted time series, multiple‐baseline, stepped wedge and any controlled nonrandomised or quasi‐experimental trial.

Abbreviations: ECEC, early childhood education and care; RCT, randomised controlled trial.

### 2.2 Stage 2: Secondary Data Analysis and Synthesis of Evidence Underpinning Practice Elements

#### 2.2.1 Step 3: Extract Practice Elements of RCTs and Map to Recommendations for ECEC

Two authors (M.L., L.G., H.T., A.R.) extracted practice elements of healthy eating and physical activity interventions evaluated in RCTs identified in Step 2, with discrepancies resolved via consensus. Practice elements were defined as specific individual components within an intervention or program that contribute to the impact of an intervention and/or as reported by the study authors (e.g., providing healthier foods in care; providing opportunity for physical activity). These practice elements were then mapped to recommendations from the list identified in Step 1 by one author (M.L.) and checked by a second author (L.G., H.T. or A.G.), with a third author (S.L.Y.) resolving discrepancies. If we were unable to map a practice element to a recommendation, we then mapped to broad practice themes. If we were unable to map practice elements to the identified recommendations nor broad practice themes, we recorded these intervention practice elements separately as ‘additional practices’.

#### 2.2.2 Step 4: Apply Framework to Assess the Evidence Underpinning Practice Elements

For each RCT, two authors (M.L., L.G. or H.T.) extracted the direction of effect on key dietary and physical activity outcomes, as outlined in a core outcome set (i.e., an agreed standardised set of outcomes that should be measured and reported) for early childhood obesity prevention [17]. For diet, this included diet quality and intake of fruit, vegetable, fruit and vegetable (combined), sugar‐sweetened beverages and discretionary food (i.e., less healthy foods that are typically high in sugar, salt and saturated fat). Physical activity outcomes included total physical activity, moderate‐to‐vigorous physical activity, sedentary behaviour and energetic movement (measured by counts and steps).

To assess the evidence underpinning practice elements, we used a vote‐counting approach consistent with Synthesis without Meta‐Analysis guidelines [18]. We counted the number of studies reporting a positive or negative effect on each child diet and physical activity outcome, based on standardised direction of effects. RCTs were included in vote‐counting for a practice element if they measured a relevant outcome, with each outcome assessed individually. RCTs could be included across multiple vote‐counting analyses (i.e., if the RCT was mapped to multiple practices and/or reported on multiple outcomes of interest).

Consistent with the approach used previously [19], we assessed each practice element (where three or more studies were available) based on the following categorisation framework:

- *Likely beneficial*: ≥ 75% of included primary studies demonstrated positive findings (regardless of significance) on the examined outcome.
- *Possibly beneficial (more evidence needed)*: 51%–74% of included primary studies demonstrated positive findings (regardless of significance) on the examined outcome.
- *Possibly not beneficial (more evidence needed)*: The majority (≥ 50%) of included primary studies demonstrated negative findings (regardless of significance) on the examined outcome.
- *Not beneficial*: All included primary studies demonstrated negative findings (regardless of significance) on the examined outcome.
- *No conclusions possible due to lack of evidence*: If ≤ 2 primary studies examined this outcome, no conclusions were able to be drawn.

#### 2.2.3 Step 5: Classify Practice Elements Within WHO Standards to Provide Practice Guidance

Lastly, we classified each practice element to one of four global standards outlined within the WHO Standards for Healthy Eating, Physical Activity, Sedentary Behaviour and Sleep in Early Childhood and Care Settings [12]. The standards include: Standard 1—‘build children's knowledge and skills’; Standard 2—‘provide supportive environments’; Standard 3—‘work with families/primary caregivers about healthy eating and movement behaviours’; and Standard 4—‘ensure safety’.

## 3 Results

### 3.1 Stage 1: Identifying the Existing Data

#### 3.1.1 Step 1: Obtain Existing Guidance to Identify Specific Recommendations for ECEC

The list of existing guidance included seven broad practice themes encompassing 36 recommendations for healthy eating and eight broad practice themes encompassing 44 recommendations for physical activity.

#### 3.1.2 Step 2: Gather RCTs Which Examine the Effectiveness of ECEC‐Based Interventions to Improve Child Diet or Physical Activity

Overall, 78 RCTs described across 80 manuscripts were obtained; 22 RCTs targeted healthy eating, 51 targeted physical activity and 7 targeted both (see Supporting Information for complete reference list of included RCTs). All included RCTs are briefly summarised in Table S1 and have been described in detail in the original review publications [10, 11, 15, 16]. The majority of RCTs (*n* = 76, 97.4%) were delivered in centre‐based ECEC and two (2.6%) were delivered in family day care services. All RCTs were conducted in high‐ or upper‐middle‐income countries, with the majority of studies conducted in the United States (*n* = 29, 37.2%) and Australia (*n* = 16, 20.5%). Sample sizes ranged from 1 to 309 services and included between 39 and 4964 children.

### 3.2 Stage 2: Secondary Data Analysis and Synthesis of Evidence Underpinning Common Practice Elements

#### 3.2.1 Step 3: Extract Practice Elements of RCTs and Map to Recommendations for ECEC

RCTs provided evidence for 26 healthy eating and 30 physical activity practice elements which could be mapped to recommendations or themes. None of the RCTs mapped to the remaining 11 healthy eating recommendations and 14 physical activity recommendations (Tables S2 and S3). We also recorded two additional practices: restructuring the scheduling of play opportunities to promote active play and parent involvement in child physical activity.

#### 3.2.2 Step 4: Apply Framework to Assess the Evidence Underpinning Practice Elements

Where a study identified from systematic reviews in Step 2 did not report on the effect of the intervention on any of the outcomes of interest, these studies were excluded from the vote‐counting approach (*n* = 9 for healthy eating [20, 21, 22, 23, 24, 25, 26, 27, 28]; *n* = 4 for physical activity [20, 21, 29, 30]).

From 29 RCTs reporting on healthy eating, we categorised 16 practice elements as likely beneficial, one as possibly beneficial and one as possibly not beneficial, based on our categorisation framework (Table S2). No conclusions could be drawn on the remaining 19 practice elements due to insufficient numbers of included RCTs that targeted these.

From 58 RCTs reporting on physical activity, we categorised 19 practice elements as likely beneficial, six as possibly beneficial and one as possibly not beneficial (Table S3). We could draw no conclusions on the remaining 20 practice elements.

#### 3.2.3 Step 5: Classify Practice Elements Within WHO Standards to Provide Practice Guidance

We classified four healthy eating practice elements within Standard 1; 24 to Standard 2; eight to Standard 3; and one to Standard 4 of the WHO Standards. We classified 16 physical activity practice elements within Standard 1; 16 to Standard 2; seven to Standard 3; and seven to Standard 4 of the WHO Standards. There were some likely beneficial practice elements included across Standards 1, 2 and 3 for both healthy eating and physical activity, and no likely beneficial practice elements for Standard 4 (see Tables S2 and S3).

## 4 Discussion

This study applied a systematic process to describe the intervention evidence‐base underpinning healthy eating and physical activity practice elements in ECEC. The majority of practice elements were categorised overall as likely beneficial, and none were categorised as not beneficial. Two additional practices (restructuring the scheduling of play opportunities to promote active play; parent involvement in child physical activity) were identified and categorised as likely beneficial. Future revision of ECEC guidelines should consider evidence from this review to support the retainment of existing recommendations and justify the development of new recommendations supported by evidence. Practice elements mapped across all four WHO standards.

All included interventions were multi‐component, as is common in public health research; therefore, the effectiveness of each practice element in isolation was not able to be directly explored. Our mapping process categorised evidence based on the proportion of RCTs that included a particular practice element and reported a positive direction of effect over all RCTs including that practice element. Therefore, the beneficial practice elements should be interpreted in the context of a multi‐component intervention rather than as a single intervention strategy. Study designs, such as factorial‐RCTs, are needed to understand the relative benefits of individual practice elements. For example, Zarnowieki and colleagues outline a multiphase optimisation strategy including a factorial‐RCT that seeks to identify the benefits of changing food provision, curriculum and supportive mealtime practices in ECEC on child's vegetable intake in isolation [31]. Such research is important to better inform the development of evidence‐based guidelines in the ECEC setting for high‐income countries.

Our mapping to the WHO Standards [12] identified several likely beneficial practice elements aligned to Standards 1, 2 and 3; however, most practice elements mapped to Standard 4 did not have sufficient evidence to allow for categorisation. These findings are not unexpected, given the majority of interventions target children's knowledge, environments and families [11, 15, 32]. Nevertheless, researchers may consider incorporating practice elements which address Standard 4 in future healthy eating and physical interventions and examining the effects on child health outcomes. While it may not be feasible to include practice elements which address all WHO Standards within a single intervention or program, these findings can provide guidance for policy makers, prevention practitioners and ECEC settings looking to prioritise and implement evidence‐based practices to promote healthy eating and physical activity in high‐income countries.

Future guideline iterations should also consider refining recommendations in line with terminology which reflects contemporary frameworks, such as more recent Australian‐endorsed guidance (e.g., the *Early Years Learning Framework v2.0* [33] and the *National Quality Standard* [34]). These frameworks now emphasise ‘energetic play’ (rather than the earlier focus on ‘structured physical activity’) recognising the importance of spontaneous, child‐led movement throughout the day. Healthy eating terminology has also evolved from prescriptive concepts such as ‘food groups’ and ‘nutrition education’ towards more holistic language promoting ‘food literacy’, ‘responsive feeding’, and ‘positive mealtime environments’. Processes to refine recommendations should also be conducted with other relevant stakeholders to ensure alignment with current terminology [9].

### 4.1 Limitations

The RCT evidence was gathered from existing reviews, with search strategies conducted between 2019 and 2022; therefore, an update of these reviews is required to capture the most recent evidence. These findings only consider the impact on child diet and physical activity outcomes. An examination of additional relevant health outcomes (e.g., sedentary behaviour, screen time, cognitive or social and emotional learning outcomes) may provide a more holistic understanding of the impact of practice elements. We used vote‐counting approaches to assess evidence underpinning practice elements, where each study was given equal weighting. Therefore, sample sizes, specific effect sizes and study characteristics did not contribute to the result. We employed a secondary data analysis; therefore, potential bias may have occurred due to relying on adequate reporting of practice elements by the original study authors. While additional practices were captured where possible, the mapping process may also have been limited by the recommendations identified in the guidelines which were published between 1999 and 2020 [8], and therefore may be outdated. Finally, the study examined universally recommended policies and practices. Recommendations or considerations specific to improve healthy eating and physical activity among priority populations in Australia, where the determinants of these behaviours (and so the effectiveness of interventions) were not explored [35, 36].

## 5 Conclusion

Investing in early interventions to promote healthy eating and activity may help alleviate the burdens of chronic disease, and has been identified as a priority [37, 38]. This study describes the evidence‐base underpinning common practice elements to promote healthy eating and physical activity in children attending ECEC services identifying key areas of support and potential for monitoring. The findings identify variability in the current evidence‐base underpinning key guideline recommended policies and practices in this setting. The study highlights the importance of future research to address key evidence gaps. One strategy to achieve this could include the embedding of evaluations as part of funded programs or improvement initiatives to support guideline implementation in this setting [39].

## Funding

This work was supported by the National Health and Medical Research Council (NHMRC) Centre for Research Excellence grant (CIA: Wolfenden, CIG Yoong APP1153479). The contents are the responsibility of the authors and do not reflect the views of the NHMRC. AG was supported by a Heart Foundation Postdoctoral Fellowship (102518). LW was supported by a NHMRC Leadership Grant (APP1197022). SLY was supported by a Heart Foundation Future Leader Fellowship (106654). Deakin University, Hunter New England Local Health District, University of Newcastle, and Hunter Medical Research Institute provided infrastructure and in‐kind support.

## Ethics Statement

Ethics approval and consent to participate are not applicable due to the use of unidentifiable secondary data.

## Conflicts of Interest

The authors declare no conflicts of interest.

## Supporting information


**Data S1:** Appendix.

## Data Availability

The data that support the findings of this study are available from the corresponding author upon reasonable request.
