# Supplementary material for: Evidence Mapping: Healthy Eating and Physical Activity Practice Elements in Early Childhood Education and Care
Source: Health Promot J Austr. 2026 Mar 12;37(2):e70170. doi: 10.1002/hpja.70170 (PMC12982912; doi:10.1002/hpja.70170)
Supplement: Supplementary file 1 — Data S1: Appendix. [file HPJA-37-0-s001.docx]

**Appendices**

References of Included Randomised Controlled Trials

1. Annesi JJ, Smith AE, Tennant GA. Effects of the Start For Life treatment on physical activity in primarily African American preschool children of ages 3-5 years*. Psychol Health Med*. 2013;18(3):300-9.

2. Annesi JJ, Smith AE, Tennant G. Cognitive-behavioural physical activity treatment in African-American pre-schoolers: effects of age, sex, and BMI. *J Paediatr Child Health*. 2013;49(2):E128-32.

3. Andersen E, Ovreas S, Jorgensen KA, Borch-Jenssen J, Moser T. Children's physical activity level and sedentary behaviour in Norwegian early childhood education and care: effects of a staff-led cluster-randomised controlled trial. *BMC Public Health*. 2020;20(1):1651.

4. Alkon A, Crowley AA, Neelon SE, Hill S, Pan Y, Nguyen V, et al. Nutrition and physical activity randomized control trial in child care centers improves knowledge, policies, and children's body mass index. *BMC Public Health*. 2014;14:215.

5. Alhassan S, Sudarsky L, Dangol G, Zhou W, Turley A, Sylvia AM, et al. Feasibility and Preliminary Efficacy of a Childcare Provider-led Activity Intervention on Toddlers' Physical Activity Levels: A Pilot Randomized Controlled Study. *Child Care Pract*. 2022;31(1):34-48.

6. Alhassan S, St. Laurent CW, Burkart S, Greever CJ, Ahmadi MN. Feasibility of Integrating Physical Activity Into Early Education Learning Standards on Preschooler’s Physical Activity Levels. *Journal of Physical Activity and Health*. 2019;16(2):101-7.

7. Alhassan S, Sirard JR, Robinson TN. The effects of increasing outdoor play time on physical activity in Latino preschool children. *International Journal of Pediatric Obesity*. 2007;2(3):153-8.

8. Alhassan S, Nwaokelemeh O, Mendoza A, Shitole S, Puleo E, Pfeiffer KA, et al. Feasibility and Effects of Short Activity Breaks for Increasing Preschool-Age Children's Physical Activity Levels*. Journal of School Health*. 2016;86(7):526-33.

9. Alhassan S, Nwaokelemeh O, Lyden K, Goldsby T, Mendoza A. A Pilot Study to Examine the Effect of Additional Structured Outdoor Playtime on Preschoolers' Physical Activity Levels. *Child Care in Practice*. 2013;19(1):23-35.

10. Alhassan S, Nwaokelemeh O, Ghazarian M, Roberts J, Mendoza A, Shitole S. Effects of locomotor skill program on minority preschoolers’ physical activity levels. Pediatric Exercise Science. 2012;24(3):435-49.

11. Aivazidis D, Venetsanou F, Aggeloussis N, Gourgoulis V, Kambas A. Enhancing Motor Competence and Physical Activity in Kindergarten. *J Phys Act Health*. 2019;16(3):184-90.

12. Afshin A, Sur PJ, Fay KA, Cornaby L, Ferrara G, Salama JS, et al. Health effects of dietary risks in 195 countries, 1990-2017: a systematic analysis for the Global Burden of Disease Study 2017. *The Lancet*. 2019;393(10184):1958-72.

13. Adamo KB, Wasenius NS, Grattan KP, Harvey ALJ, Naylor P-J, Barrowman NJ, et al. Effects of a Preschool Intervention on Physical Activity and Body Composition. *J Pediatr*. 2017;188:42-9.e2.

14. Başkale H, Bahar Z. Outcomes of nutrition knowledge and healthy food choices in 5-to 6-year-old children who received a nutrition intervention based on Piaget’s theory. *J Spec Pediatr Nurs*. 2011;16(4):263-79.

15. Blomkvist EAM, Wills AK, Helland SH, Hillesund ER, Øverby NC. Effectiveness of a kindergarten-based intervention to increase vegetable intake and reduce food neophobia amongst 1-year-old children: a cluster randomised controlled trial. *Food & Nutrition Research*. 2021;65.

16. Bonis M, Loftin M, Ward D, Tseng TS, Clesi A, Sothern M. Improving physical activity in daycare interventions. *Childhood Obesity*. 2014;10(4):334-41.

17. Bonvin A, Barral J, Kakebeeke TH, Kriemler S, Longchamp A, Schindler C, et al. Effect of a governmentally-led physical activity program on motor skills in young children attending child care centers: a cluster randomized controlled trial. *International journal of behavioral nutrition and physical activity*. 2013;10:1-12.

18. Byun W, Lau EY, Brusseau TA. Feasibility and Effectiveness of a Wearable Technology-Based Physical Activity Intervention in Preschoolers: A Pilot Study. *Int J Environ Res Public Health*. 2018;15(9):23

19. Cardon G, Labarque V, Smits D, Bourdeaudhuij ID. Promoting physical activity at the pre-school playground: The effects of providing markings and play equipment. *Prev Med*. 2009;48(4):335-40.

20. Chow AF, Leis A, Humbert L, Muhajarine N, Engler-Stringer R. Healthy start – Départ santé: A pilot study of a multilevel intervention to increase physical activity, fundamental movement skills and healthy eating in rural childcare centres. *Can J Public Health*. 2016;107(3):e312-e8.

21. De Bock F, Breitenstein L, Fischer JE. Positive impact of a pre-school-based nutritional intervention on children's fruit and vegetable intake: results of a cluster-randomized trial. *Public Health Nutr*. 2012;15(3):466-75.

22. De Bock F, Genser B, Raat H, Fischer JE, Renz-Polster H. A Participatory Physical Activity Intervention in Preschools: A Cluster Randomized Controlled Trial. *Am J Prev Med*. 2013;45(1):64-74.

23. De Coen V, De Bourdeaudhuij I, Vereecken C, Verbestel V, Haerens L, Huybrechts I, et al. Effects of a 2-year healthy eating and physical activity intervention for 3–6-year-olds in communities of high and low socio-economic status: the POP (Prevention of Overweight among Pre-school and school children) project. *Public Health Nutr*. 2012;15(9):1737-45.

24. Driediger M, Truelove S, Johnson AM, Vanderloo LM, Timmons BW, Burke SM, et al. The Impact of Shorter, More Frequent Outdoor Play Periods on Preschoolers' Physical Activity during Childcare: A Cluster Randomized Controlled Trial. *Int J Environ Res Public Health*. 2019;16(21):26.

25. Eliakim A, Nemet D, Balakirski Y, Epstein Y. The effects of nutritional-physical activity school-based intervention on fatness and fitness in preschool children. *J Pediatr Endocrinol Metab*. 2007;20(6):711-8.

26. Ellis YG, Cliff DP, Howard SJ, Okely AD. Feasibility, acceptability, and potential efficacy of a childcare-based intervention to reduce sitting time among pre-schoolers: A pilot randomised controlled trial. *J Sports Sci*. 2019;37(2):146-55.

27. Engel A, Broderick C, Hardy L, Ward R, van Doorn N, Kwai N, et al. Effect of a Fundamental Motor Skills Intervention on Fundamental Motor Skill and Physical Activity in a Preschool Setting: A Cluster Randomized Controlled Trial. *Pediatric Exerc Sci*. 2021:1-10.

28. Finch M, Wolfenden L, Morgan PJ, Freund M, Jones J, Wiggers J. A cluster randomized trial of a multi-level intervention, delivered by service staff, to increase physical activity of children attending center-based childcare. *Prev Med*. 2014;58:9-16.

29. Fitzgibbon ML, Stolley MR, Schiffer LA, Braunschweig CL, Gomez SL, van Horn L, et al. Hip-Hop to Health Jr. Obesity Prevention Effectiveness Trial: Postintervention Results. *Obesity*. 2011;19(5):994-1003.

31. Fitzgibbon ML, Stolley MR, Schiffer L, Kong A, Braunschweig CL, Gomez-Perez SL, et al. Family-based hip-hop to health: outcome results. *Obesity*. 2013;21(2):274-83.

32. Gans KM, Tovar A, Kang A, Ward DS, Stowers KC, von Ash T, et al. A multi-component tailored intervention in family childcare homes improves diet quality and sedentary behavior of preschool children compared to an attention control: results from the Healthy Start-Comienzos Sanos cluster randomized trial. *Int J Behav Nutr Phys Act*. 2022;19(1):45.

33. Goldfield GS, Harvey ALJ, Grattan KP, Temple V, Naylor P-J, Alberga AS, et al. Effects of Child Care Intervention on Physical Activity and Body Composition. *Am J Prev Med*. 2016;51(2):225-31.

34. Grummon AH, Cabana MD, Hecht AA, Alkon A, McCulloch CE, Brindis CD, et al. Effects of a multipronged beverage intervention on young children's beverage intake and weight: A cluster-randomized pilot study. *Public Health Nutr*. 2019;22(15):2856-67.

35. Hoffman JA, Schmidt EM, Arguello DJ, Eyllon MN, Castaneda-Sceppa C, Cloutier G, et al. Online preschool teacher training to promote physical activity in young children: A pilot cluster randomized controlled trial. *Sch Psychol*. 2020;35(2):118-27.

36. Iaia M, Pasini M, Burnazzi A, Vitali P, Allara E, Farneti M. An educational intervention to promote healthy lifestyles in preschool children: a cluster-RCT. *Int J Obes*. 2017;41(4):582-90.

37. Jones RA, Riethmuller A, Hesketh K, Trezise J, Batterham M, Okely AD. Promoting fundamental movement skill development and physical activity in early childhood settings: a cluster randomized controlled trial. *Pediatric Exerc Sci*. 2011;23(4):600-15.

38. Jones J, Wyse R, Finch M, Lecathelinais C, Wiggers J, Marshall J, et al. Effectiveness of an intervention to facilitate the implementation of healthy eating and physical activity policies and practices in childcare services: a randomised controlled trial. *Implement Sci*. 2015;10:1-15.

39. Jones RA, Okely AD, Hinkley T, Batterham M, Burke C. Promoting gross motor skills and physical activity in childcare: A translational randomized controlled trial. *J Sci Med Sport*. 2016;19(9):744-9.

40. Kipping R, Langford R, Brockman R, Wells S, Metcalfe C, Papadaki A, et al. Child-care self-assessment to improve physical activity, oral health and nutrition for 2-to 4-year-olds: a feasibility cluster RCT. *Public Health Res*. 2019;7(13):1-164.

41. Kobel S, Wartha O, Lämmle C, Dreyhaupt J, Steinacker JM. Intervention effects of a kindergarten-based health promotion programme on obesity related behavioural outcomes and BMI percentiles. *Prev Med Rep*. 2019;15:100931.

42. Kornilaki EN, Skouteris H, Morris H. Developing connections between healthy living and environmental sustainability concepts in Cretan preschool children: a randomized trial. *Early Child Dev Care*. 2022;192(11):1685-98.

43. Kristiansen AL, Bjelland M, Himberg-Sundet A, Lien N, Holst R, Frost Andersen L. Effects of a cluster randomized controlled kindergarten-based intervention trial on vegetable consumption among Norwegian 3–5-year-olds: the BRA-study. *BMC Public Health*. 2019;19(1):1098.

44. Leis A, Ward S, Vatanparast H, Humbert ML, Chow AF, Muhajarine N, et al. Effectiveness of the Healthy Start-Départ Santé approach on physical activity, healthy eating and fundamental movement skills of preschoolers attending childcare centres: a randomized controlled trial. *BMC Public Health*. 2020;20(1):1-12.

45. Lumeng JC, Miller AL, Horodynski MA, Brophy-Herb HE, Contreras D, Lee H, et al. Improving self-regulation for obesity prevention in head start: a randomized controlled trial. *Pediatrics*. 2017;139(5).

46. Malden S, Reilly JJ, Gibson AM, Bardid F, Summerbell C, De Craemer M, et al. A feasibility cluster randomised controlled trial of a preschool obesity prevention intervention: toyBox-Scotland. *Pilot Feasibility Stud*. 2019;5(1).

47. Mavilidi MF, Rigoutsos S, Venetsanou F. Training Early Childhood Educators to Promote Children's Physical Activity. *Early Child Educ J*. 2021;50:785-94.

48. Mazzucca SL. Physical Activity and Sedentary Behavior in Early Care and Education Centers: Identifying Opportunities and Testing Strategies to Support Active Classroom Environments. Ann Arbor: The University of North Carolina at Chapel Hill; 2017.

49. Mehtälä MA, Sääkslahti A, Soini A, Tammelin T, Kulmala J, Villberg J, et al. The effect of the cluster randomized HIPPA intervention on childcare children's overall physical activity. *Balt J Health Phys Act*. 2017;9(4):89-111.

50. Morris H, Edwards S, Cutter-Mackenzie A, Rutherford L, Williams-Smith J, Skouteris H. Evaluating the impact of teacher-designed, wellbeing and sustainability play-based learning experiences on young children's knowledge connections: A randomised trial. *Australas J Early Child*. 2018;43(4):33-42.

51. Namenek Brouwer RJ, Benjamin Neelon SE. Watch Me Grow: A garden-based pilot intervention to increase vegetable and fruit intake in preschoolers. *BMC Public Health*. 2013;13:1-6.

52. Natale RA, Messiah SE, Asfour L, Uhlhorn SB, Delamater A, Arheart KL. Role Modeling as an Early Childhood Obesity Prevention Strategy: Effect of Parents and Teachers on Preschool Children's Healthy Lifestyle Habits. *J DevBehav Pediatr*. 2014;35(6).

53. Natale RA, Atem F, Weerakoon S, Lebron C, Mathew MS, Sardinas K, et al. An Implementation Approach Comparison of a Child Care Center-Based Obesity Prevention Program. *J Dev Behav Pediatr*. 2021;42(2):135-45.

54. O’Dwyer M, Fairclough SJ, Ridgers N, Knowles Z, Foweather L, Stratton G. Effect of a school-based active play intervention on sedentary time and physical activity in preschool children. *Health Educ Res*. 2013;28(6):931-42.

55. Okely AD, Stanley RM, Jones RA, Cliff DP, Trost SG, Berthelsen D, et al. ‘Jump start’ childcare-based intervention to promote physical activity in pre-schoolers: six-month findings from a cluster randomised trial. *Int J Behav Nutr Phys Act*. 2020;17(1):6.

56. Palmer KK, Chinn KM, Robinson LE. The effect of the CHAMP intervention on fundamental motor skills and outdoor physical activity in preschoolers. *J Sport Health Sci*. 2019;8(2):98‐105.

57. Pate RR, Brown WH, Pfeiffer KA, Howie EK, Saunders RP, Addy CL, et al. An Intervention to Increase Physical Activity in Children: A Randomized Controlled Trial With 4-Year-Olds in Preschools*. Am J Prev Med*. 2016;51(1):12-22.

58. Pearson N, Finch M, Sutherland R, Kingsland M, Wolfenden L, Wedesweiler T, et al. An mHealth Intervention to Reduce the Packing of Discretionary Foods in Children’s Lunch Boxes in Early Childhood Education and Care Services: Cluster Randomized Controlled Trial. *J Med Internet Res*. 2022;24(3):e27760.

59. Peden M, Eady M, Okely A, Patterson KM, Jones R. A blended professional learning intervention for early childhood educators to target the promotion of physical activity and healthy eating: the HOPPEL cluster randomized stepped-wedge trial. *BMC Public Health*. 2022;22:1.

60. De Craemer M, Verloigne M, De Bourdeaudhuij I, Androutsos O, Iotova V, Moreno L, et al. Effect and process evaluation of a kindergarten-based, family-involved cluster randomised controlled trial in six European countries on four-to six-year-old children’s steps per day: the ToyBox-study. I*nt J Behav Nutr Phys Act*. 2017;14(1):1-16./ De Craemer M, Verbestel V, Verloigne M, Androutsos O, Moreno L, Iotova V, et al. Combining effect and process evaluation on european preschool children’s snacking behavior in a kindergarten-based, family-involved cluster randomized controlled trial: the toybox study. *Int J Environ Res Public Health*. 2020;17(19):7312.

61. Puder JJ, Marques-Vidal P, Schindler C, Zahner L, Niederer I, Bürgi F, et al. Effect of multidimensional lifestyle intervention on fitness and adiposity in predominantly migrant preschool children (Ballabeina): cluster randomised controlled trial. *BMJ*. 2011;13:343.

62. Ray C, Figuereido R, Vepsäläinen H, Lehto R, Pajulahti R, Skaffari E, et al. Effects of the preschool-based family-involving DAGIS intervention program on Children’s energy balance-related behaviors and self-regulation skills: a clustered randomized controlled trial. *Nutrients*. 2020;12(9):2599.

63. Razak LA, Yoong SL, Wiggers J, Morgan PJ, Jones J, Finch M, et al. Impact of scheduling multiple outdoor free-play periods in childcare on child moderate-to-vigorous physical activity: a cluster randomised trial. *Int J Behav Nutr Phys Act*. 2018;15(1):34.

64. Reilly JJ, Kelly L, Montgomery C, Williamson A, Fisher A, McColl JH, et al. Physical activity to prevent obesity in young children: cluster randomised controlled trial. *BMJ*. 2006;333(7577):1041.

65. Roberts-Gray C, Ranjit N, Sweitzer SJ, Byrd-Williams CE, Romo-Palafox MJ, Briley ME, et al. Parent packs, child eats: Surprising results of Lunch is in the Bag‘s efficacy trial. *Appetite*. 2018;121:249-62.

66. Roth K, Kriemler S, Lehmacher W, Ruf KC, Graf C, Hebestreit H. Effects of a Physical Activity Intervention in Preschool Children. *Med Sci Sports Exerc*. 2015;47(12):2542-51.

67. Schmidt M, Mavilidi MF, Singh A, Englert C. Combining physical and cognitive training to improve kindergarten children’s executive functions: A cluster randomized controlled trial. *Contemp Educ Psychol*. 2020;63:101908.

68. Seward K, Wolfenden L, Finch M, Wiggers J, Wyse R, Jones J, et al. Improving the implementation of nutrition guidelines in childcare centres improves child dietary intake: findings of a randomised trial of an implementation intervention. *Public Health Nutr*. 2018;21(3):607-17.

69. Sharp CA. Development and Evaluation of a Healthy Eating and Physical Activity Behaviour Change Intervention Targeting 3-4 year old Children at School, Extending to the Home. Ann Arbor: Bangor University (United Kingdom); 2017.

70. Szpunar M, Driediger M, Johnson AM, Vanderloo LM, Burke SM, Irwin JD, et al. Impact of the Childcare Physical Activity (PLAY) Policy on Young Children's Physical Activity and Sedentary Time: A Pilot Clustered Randomized Controlled Trial. *Int J Environ Res Public Health*. 2021;18(14):13.

71. Telford RM, Olive LS, Telford RD. A peer coach intervention in childcare centres enhances early childhood physical activity: The Active Early Learning (AEL) cluster randomised controlled trial. *Int J Behav Nutr Phys Act*. 2021;18(1):37.

72. Trost SG, Fees B, Dzewaltowski D. Feasibility and efficacy of a “move and learn” physical activity curriculum in preschool children. *J Phys Act Health*. 2008;5(1):88-103.

73. Tucker P, Vanderloo LM, Johnson AM, Burke SM, Irwin JD, Gaston A, et al. Impact of the Supporting Physical Activity in the Childcare Environment (SPACE) intervention on preschoolers' physical activity levels and sedentary time: a single-blind cluster randomized controlled trial. *Int J Behav Nutri Phys Act*. 2017;14(1):120.

74. Vaughn AE, Hennink-Kaminski H, Moore R, Burney R, Chittams JL, Parker P, et al. Evaluating a child care-based social marketing approach for improving children’s diet and physical activity: results from the Healthy Me, Healthy We cluster-randomized controlled trial. *Transl Behav Med*. 2021;11(3):775-84.

75. Vereecken C, Huybrechts I, Van Houte H, Martens V, Wittebroodt I, Maes L. Results from a dietary intervention study in preschools “Beastly Healthy at School”. *Int J Public Health*. 2009;54:142-9.

76. Wadsworth DD, Johnson JL, Carroll AV, Pangelinan MM, Rudisill ME, Sassi J. Intervention Strategies to Elicit MVPA in Preschoolers during Outdoor Play. *Int J Environ Res Public Health*. 2020;17(2):19.

77. Ward DS, Vaughn AE, Burney RV, Hales D, Benjamin-Neelon SE, Tovar A, et al. Keys to healthy family child care homes: Results from a cluster randomized trial. *Prev Med*. 2020;132:105974.

78. Witt KE, Dunn C. Increasing fruit and vegetable consumption among preschoolers: evaluation of color me healthy. *J Nutr Educ Behav*. 2012;44(2):107-13.

79. Wolfenden L, Jones J, Parmenter B, Razak LA, Wiggers J, Morgan PJ, et al. Efficacy of a free-play intervention to increase physical activity during childcare: a randomized controlled trial. *Health Educ Res*. 2019;34(1):84-97.

80. Yoong SL, Grady A, Wiggers JH, Stacey FG, Rissel C, Flood V, et al. Child-level evaluation of a web-based intervention to improve dietary guideline implementation in childcare centers: a cluster-randomized controlled trial. *Am J Clin Nutr*. 2020;111(4):854-63.

**Supplementary Table 1.** Characteristics of randomized controlled trials included in vote-counting

| **Author name, year** | **Country** | **Settings** | **Sample size** | **Target risk factor** | **Outcomes assessed** | **Mapping practice elements to WHO Standards^a^ and practice recommendations^b^** |
| --- | --- | --- | --- | --- | --- | --- |
| Adamo, 2017 | Canada | Centre-based + home | 18 services (215 children) | Physical activity | MVPA, total physical activity, sedentary behaviour, counts, steps | Physical activity  Standard 1: 1.1; 1.2; 1.3; 1.4; 1.5; 1.7; 4.4  Standard 2: 1.6; 3.3; 3.4; 4.1; 4.3; 4.5; 4.6; 8.1; 8.2; 8.3  Standard 3: 6.8; Parent/caregiver involvement in child physical activity |
| Aivazidis, 2019 | Greece | Centre-based | 4 services (140 children) | Physical activity | Steps | Physical activity  Standard 1: 1.1; 1.4; 1.5; 1.6; 1.7  Standard 2: 3.3;3.4; 4.3  Standard 3: Parent/caregiver involvement in child physical activity |
| Alhassan, 2007 | USA | Centre-based | 1 service (33 children) | Physical activity | MVPA, sedentary behaviour, counts | Physical activity  Standard 1: 1.5 |
| Alhassan, 2012 | USA | Centre-based | 2 services (78 children) | Physical activity | MVPA, sedentary behaviour | Physical activity  Standard 1: 1.1; 1.3; 1.4; 1.6; 1.7  Standard 2: 3.4; 4.1; 4.2; 4.3 |
| Alhassan, 2013 | USA | Centre-based | 2 services (75 children) | Physical activity | MVPA, sedentary behaviour | Physical activity  Standard 1: 1.1; 1.4  Standard 2: 4.1; 4.2; 4.3; 8.1 |
| Alhassan, 2016 | USA | Centre-based | 10 services (291 children) | Physical activity | MVPA, sedentary behaviour | Physical activity  Standard 1: 1.1; 1.3; 1.4; 1.7  Standard 2: 4.3 |
| Alhassan, 2019 | USA | Centre-based + home | 2 services (52 children) | Physical activity | MVPA, sedentary behaviour | Physical activity  Standard 1: 1.1; 1.3; 1.4; 7.1  Standard 2: 3.4; 4.3  Standard 3: 2.3; 6.8; Parent/caregiver involvement in child physical activity |
| Alhassan, 2022 | USA | Centre-based | 3 services (50 children) | Physical activity | MVPA, sedentary behaviour, steps | Physical activity  Standard 1: 1.1; 1.3; 1.4  Standard 2: 3.4; 4.2 |
| Alkon, 2014 | USA | Centre-based + home | 18 services (552 children) | Physical activity | Sedentary behaviour | Physical activity  Standard 1: 1.4; 2.2  Standard 2: 2.4; 3.3; 3.4  Standard 3: 3.1; Parent/caregiver involvement in child physical activity |
| Anderson, 2020 | Norway | Centre-based | 11 services (116 children) | Physical activity | MVPA, total physical activity, sedentary behaviour, steps | Physical activity  Standard 1: 1.1; 2.2  Standard 2: 3.3; 3.4; 8.1  Standard 3: Parent/caregiver involvement in child physical activity |
| Annesi, 2013a | USA | Centre-based | 32 classes (275 children) | Physical activity | MVPA, sedentary behaviour | Physical activity:  Standard 1: 1.1; 1.3; 1.4; 1.7; 1.2  Standard 2:3.4; 4.2 |
| Annesi, 2013b | USA | Centre based | 19 classes (338 children) | Physical activity | MVPA, sedentary behaviour | Physical activity  Standard 1: 1.1; 1.2; 1.3; 1.4; 1.7  Standard 2: 3.4; 4.2 |
| Baskale, 2011 | Turkey | Centre-based | 12 services (238 children) | Healthy eating | Vegetable consumption | Healthy eating  Standard 1: 3.1 |
| Blomkvist, 2021 | Norway | Centre-based | 46 services (267 children) | Healthy eating | Vegetable consumption | Healthy eating  Standard 1: 3.1  Standard 2: 1.2; 4.3; 5.6; 5.7; 6.3; 6.4  Standard 3: 7.3; 7.5 |
| Bonis, 2014 | USA | Centre-based + home | 26 services (209 children) | Physical activity | MVPA, total physical activity, sedentary behaviour | Physical activity  Standard 1: 2.2  Standard 2: 3.3; 3.4  Standard 3: Parent/caregiver involvement in child physical activity |
| Bonvin, 2013 | Switzerland | Centre-based + home | 58 services (648 children) | Physical activity | MVPA, total physical activity | Physical activity  Standard 1: 1.1; 1.7  Standard 2: 3.3; 3.4; 4.2; 8.1; 8.2; 8.3  Standard 3: 3.1; Parent/caregiver involvement in child physical activity |
| Byun, 2018 | USA | Centre-based | 5 services (115 children) | Physical activity | MVPA, total physical activity, sedentary behaviour | Physical activity  Standard 1: 1.1; 1.2; 1.6; 5.1  Standard 2: 3.3; 4.2; 4.3; 5.2; 5.3 |
| Cardon, 2009 | Belgium | Centre-based | 40 services (634 children) | Physical activity | Total physical activity, sedentary behaviour | Physical activity  Standard 2: 8.1 |
| Chow, 2016 | Canada | Centre-based | 6 services (69 children) | Physical activity | MVPA, total physical activity, sedentary behaviour, counts | Physical activity  Standard 1: 1.1; 1.7  Standard 2: 3.4; 4.2  Standard 3: 3.1 |
| De Bock, 2012 | Germany | Centre-based + home | 18 services (377 children) | Healthy eating | Fruit consumption, vegetable consumption | Healthy eating  Standard 1: 3.1; 5.4  Standard 2: 6.3  Standard 3: 7.2; 7.5; 7.6 |
| De Bock, 2013 | Germany | Centre-based + home + wider community | 39 services (826 children) | Physical activity | MVPA, sedentary behaviour, counts | Physical activity:  Standard 1: 1.1  Standard 2: 3.4  Standard 3: 3.1; Parent/caregiver involvement in child physical activity |
| De Coen, 2012 | Belgium | Centre-based + wider community | 31 services (1589 children) | Healthy eating | Fruit consumption, vegetable consumption, non-core food consumption | Healthy eating  Standard 1: 3.1; 4.1  Standard 2: 1.2; 1.3; 1.4; 2; 2.1; 2.3;  Standard 3: 2.4; 7.4; 7.5; 7.6 |
| Driediger, 2019 | Canada | Centre-based | 12 services (185 children) | Physical activity | MVPA, total physical activity, sedentary behaviour | Physical activity  Standard 1: 1.2; 1.5  Standard 2: 8.6; Restructuring the scheduling of play opportunities to promote active play |
| Eliakim, 2007 | Israel | Centre-based + home | 4 services (101 children) | Physical activity | Steps | Physical activity  Standard 1: 1.1; 1.4; 1.7; 5.1  Standard 2: 4.2 |
| Ellis, 2019 | Australia | Centre-based | 4 services (115 children) | Physical activity | Sedentary behaviour, steps | Physical activity  Standard 1: 1.1;1.4; 4.4; 5.1  Standard 2: 3.3; 3.4; 4.1; 4.3; |
| Engel, 2022 | Australia | Centre-based | 4 services (50 children) | Physical activity | MVPA, total physical activity, sedentary behaviour, | Physical activity  Standard 1: 1.1; 1.4; 1.6; 1.7  Standard 2: 4.2 |
| Finch, 2014 | Australia | Centre-based | 20 services (459 children) | Physical activity | Steps | Physical activity  Standard 1: 1.1; 1.4; 1.5; 1.7; 2.2; 4.4; 5.1; 6.2  Standard 2: 2.4; 3.4; 4.1; 4.3; 8.1; 8.2 |
| Fitzgibbon, 2011 | USA | Centre-based + home | 18 services (618 children) | Healthy eating  Physical activity | Diet quality, fruit consumption, vegetable consumption  MVPA, counts | Healthy eating  Standard 1: 3.1; 4.1  Standard 3: 7.2; 7.5; 7.6  Physical activity:  Standard 1: 1.1; 1.4  Standard 2: 3.4; 4.2  Standard 3: Parent/caregiver involvement in child physical activity  Standard 4: 1.8 |
| Fitzgibbon, 2013 | USA | Centre-based + home | 4 services (147 children) | Healthy eating  Physical activity | Fruit consumption, vegetable consumption  MVPA, sedentary behaviour, counts | Healthy eating  Standard 1: 3.1  Standard 3: 7.5; 7.6  Physical activity  Standard 1: 1.1;1.4  Standard 3: Parent/caregiver involvement in child physical activity  Standard 4: 1.8 |
| Gans, 2022 | USA | Family day care | 119 services (377 children) | Healthy eating | Diet quality | Healthy eating  Standard 1: 4.1  Standard 2: 4.3 |
| Goldfield, 2016 | Canada | Centre-based | 6 services (83 children) | Physical activity | MVPA, total physical activity, sedentary behaviour | Physical activity  Standard 1: 1.1; 1.2; 1.3; 1.4; 1.5; 1.6; 1.7; 5.1  Standard 2: 3.3; 3.4; 4.1; 8.1; 8.2; 8.3 |
| Grummon, 2019 | USA | Centre-based + home | 4 services (161 children) | Healthy eating | Sugar-sweetened beverage consumption | Healthy eating  Standard 1: 3.1; 6.2  Standard 2: 1.1; 2.3; 6.2  Standard 3: 2.4; 7.2; 7.5; 7.6 |
| Hoffman, 2020 | USA | Centre-based | 6 services (58 children) | Physical activity | MVPA | Physical activity  Standard 1: 1.2; 1.4; 1.5; 1.7; 2.2  Standard 2: 3.3; 3.4; 4.1; 4.2; 8.1 |
| Iaia, 2017 | Italy | Centre-based + local health centres | 16 services (389 children) | Healthy eating | Fruit and vegetables consumption, sugar-sweetened beverage consumption | Healthy eating  Standard 1: 3.1; 5.4  Standard 2: 1.4; 2; 4.3; 5.7; 6.3; 6.4  Standard 3: 7.5 |
| Jones, 2011 | Australia | Centre-based | 2 services (97 children) | Physical activity | MVPA, sedentary behaviour, counts | Physical activity  Standard 1: 1.1; 1.4; 1.5; 1.7  Standard 2: 3.3; 4.2; 8.2 |
| Jones, 2015 | Australia | Centre-based | 128 services (NR) | Healthy eating  Physical activity | Fruit consumption vegetable consumption, non-core food consumption  MVPA, sedentary behaviour | Healthy eating  Standard 2: 2; 2.3; 4.3; 5.6  Standard 3: 2.4; 7.1  Physical activity  Standard 1: 1.1; 1.4; 1.7; 2.2; 6.2  Standard 2: 3.4; 4.1 |
| Jones, 2016 | Australia | Centre-based | 4 services (166 children) | Physical activity | MVPA, total physical activity, sedentary behaviour | Physical activity  Standard 1: 1.1; 1.4; 1.5; 1.7  Standard 2: 3.3; 3.4 |
| Kipping, 2019 | United Kingdom | Centre-based + home | 12 services (167 children) | Physical activity | MVPA, total physical activity, sedentary behaviour, counts | Physical activity  Standard 1: 1.1; 1.4; 5.1; 6.2  Standard 2: 2.4  Standard 3: 2.3; 6.8; 6.x |
| Kobel, 2019 | Germany | Centre-based + home | 57 services (973 children) | Healthy eating | Fruit and vegetable consumption, sugar-sweetened beverage consumption | Healthy eating  Standard 1: 3.1; 4.1  Standard 3: 7.2; 7.5; 7.6 |
| Kornilaki, 2021 | Greece | Centre-based | 15 services (329 children) | Healthy eating | Fruit consumption, vegetable consumption, fruit and vegetable consumption, non-core food consumption, sugar-sweetened beverage consumption | Healthy eating  Standard 1: 3.1; 4.1 |
| Kristiansen, 2019 | Norway | Centre-based + home | 73 services (663 children) | Healthy eating | Vegetable consumption | Healthy eating  Standard 1: 3.1  Standard 2: 1.2; 4.3; 5.6; 6.7  Standard 3: 7.2; 7.5; 7.6 |
| Leis, 2020 | Canada | Centre-based | 61 services (897 children) | Healthy eating  Physical activity | Fruit and vegetable consumption  MVPA, total physical activity, sedentary behaviour | Healthy eating  Standard 2: 4.3  Standard 3: 7.5  Physical activity:  Standard 1: 1.1; 1.7  Standard 2: 3.3; 3.4; 8.1 |
| Lumeng, 2017 | USA | Centre-based + home | 3 services (697 children) | Healthy eating | Fruit consumption, vegetable consumption, sugar-sweetened beverage consumption | Healthy eating  Standard 3: 7.6 |
| Malden, 2019 | Scotland | Centre-based + home | 6 services (42 children) | Physical activity | Total physical activity, sedentary behaviour, steps | Physical activity  Standard 1: 1.1; 1.7; 5.1  Standard 2: 3.4; 8.3  Standard 3: Parent/caregiver involvement in child physical activity |
| Malvilidi, 2022 | Greece | Centre-based | 7 services (150 children) | Physical activity | Steps | Physical activity  Standard 1: 1.1; 1.4; 1.5; 4.4  Standard 2: 3.3; 3.4 |
| Mazzuca, 2017 | USA | Centre-based | 26 services (182 children) | Physical activity | MVPA, total physical activity, sedentary behaviour, counts | Physical activity  Standard 1: 1.1; 1.4; 4.4  Standard 2: 3.4; 8.2 |
| Mehtala, 2017 | Finland | Centre-based + home | 14 services (102 children) | Physical activity | MVPA, total physical activity, counts | Physical activity  Standard 1: 1.1; 1.4; 1.5; 1.6; 1.7  Standard 2: 3.3; 3.4; 8.1; 8.2  Standard 3: 2.3; Parent/caregiver involvement in child physical activity |
| Morris, 2018 | Australia | Centre-based | NR (300 children) | Healthy eating | Fruit consumption, vegetable consumption, fruit and vegetable consumption, non-core food consumption, sugar-sweetened beverage consumption | Healthy eating  Standard 1: 3.1; 4.1 |
| Namenek Brouwer, 2013 | USA | Centre-based | 4 services (NR) | Healthy eating | Fruit consumption, vegetable consumption | Healthy eating  Standard 1: 3.1; 4.1; 5.4  Standard 2: 1.2; 1.3; 4.3;5.6; 5.7; 6.6; 6.7  Standard 3: 7.2 |
| Natale, 2014 | USA | Centre-based | 28 services (1211 children) | Healthy eating | Fruit consumption, vegetable consumption, non-core food consumption | Healthy eating  Standard 1: 3.1; 4.1  Standard 2: 2; 2.1; 5.6  Standard 3: 7.5; 7.6 |
| Natale, 2021 | USA | Centre-based | 24 services (825 children) | Healthy eating | Fruit consumption, vegetable consumption, non-core food consumption | Healthy eating  Standard 1: 3.1; 4.1  Standard 2: 2; 4.3; 5.7  Standard 3: 2.4 |
| O’Dwyer, 2013 | United Kingdom | Centre-based | 12 services (240 children) | Physical activity | MVPA, sedentary behaviour | Physical activity  Standard 1: 1.1; 1.3; 1.4; 2.2; 5.1 |
| Okely, 2020 | Australia | Centre-based + home | 43 services (658 children) | Physical activity | MVPA, total physical activity, sedentary behaviour | Physical activity  Standard 1: 1.1; 1.4; 1.7; 4.4; 5.1  Standard 2: 3.3; 3.4; 4.2; 4.3;  Standard 3: Parent/caregiver involvement in child physical activity |
| Palmer, 2019 | USA | Centre-based | 1 service (102 children) | Physical activity | MVPA, total physical activity, sedentary behaviour | Physical activity  Standard 1: 1.1; 1.4; 1.7 |
| Pate, 2015 | USA | Centre-based | 16 services (488 children) | Physical activity | MVPA, total physical activity, sedentary behaviour | Physical activity  Standard 1: 1.1; 1.3; 1.4; 1.5; 1.6; 1.7; 4.4  Standard 2: 3.4; 4.1; 4.2; 4.3; 8.1; 8.3 |
| Pearson, 2022 | Australia | Centre-based + home | 17 services (400 children) | Healthy eating | Fruit consumption, vegetable consumption, non-core food consumption | Healthy eating  Standard 2: 2.3  Standard 3: 7.1; 7.5 |
| Peden, 2022 | Australia | Centre-based + home | 15 services (314 children) | Physical activity | Total physical activity, sedentary behaviour | Physical activity  Standard 1: 1.4; 1.5; 1.6; 5.1  Standard 2: 2.4 3.3; 3.4; 8.3 |
| Pinket, 2016/De Craemer 2020/De Craemer 2017 | 6 European countries (Belgium, Bulgaria, Germany, Greece, Poland and Spain) | Centre-based | 309 services (4964 children) | Healthy eating  Physical activity | Fruit consumption  Steps | Healthy eating  Standard 1: 3.1; 4.1; 6.5  Standard 2: 1.4; 4.3; 5.6; 6.6  Standard 3: 7.5; 7.6  Physical activity  Standard 1: 1.1  Standard 2: 3.4; 8.1; 8.2  Standard 3: Parent/caregiver involvement in child physical activity |
| Puder, 2011 | Switzerland | Centre-based + home | 40 services (655 children) | Physical activity | Counts | Physical activity  Standard 1: 1.1; 1.4; 1.5  Standard 2: 3.4; 4.2; 4.3; 8.1; 8.2; 8.3  Standard 3: 2.3; 6.8; Parent/caregiver involvement in child physical activity  Standard 4: 1.8 |
| Ray, 2020 | Finland | Centre-based | 32 services (802 children) | Healthy eating  Physical activity | Fruit and vegetable consumption  Total physical activity | Healthy eating  Standard 1: 3.1; 4.1  Standard 2: 6.6; 6.7  Standard 3: 7.2; 7.5  Physical activity  Standard 1: 1.1  Standard 2: 3.4  Standard 3: 2.3; 6.8; Parent/caregiver involvement in child physical activity |
| Razak, 2018 | Australia | Centre-based | 10 services (439 children) | Physical activity | MVPA, total physical activity, counts | Physical activity  Standard 1: 1.1; 1.5; 1.6; 2.2  Standard 2: 3.4; 8.6; Restructuring the scheduling of play opportunities to promote active play |
| Reilly, 2006 | Scotland | Centre-based + home | 36 services (545 children) | Physical activity | MVPA, sedentary behaviour, counts | Physical activity  Standard 1: 1.1; 1.7; 6.2  Standard 3: 6.8; Parent/caregiver involvement in child physical activity |
| Roberts-Gray, 2018 | USA | Centre-based + home | 30 services (633 children) | Healthy eating | Fruit consumption, vegetable consumption | Healthy eating  Standard 1: 3.1; 4.1  Standard 3: 7.1; 7.5; 7.6 |
| Roth, 2015 | Germany | Centre-based + home | 41 services (709 children) | Physical activity | MVPA | Physical activity  Standard 1: 1.1; 1.4  Standard 2: 4.2  Standard 3: 2.3; 6.8; Parent/caregiver involvement in child physical activity |
| Schmidt, 2020 | Switzerland | Centre-based | 14 services (189 children) | Physical activity | Steps | Physical activity  Standard 1: 1.4; 1.7; 1.7; 4.4  Standard 2: 3.4 |
| Seward, 2018 | Australia | Centre-based | 45 services (243 children) | Healthy eating | Diet quality, fruit consumption, vegetable consumption, non-core food consumption | Healthy eating  Standard 2: 1.1; 1.3; 2; 2.1; 2.3; 4.3 |
| Sharp, 2017 | Wales | Centre-based + home | 4 services (172 children) | Physical activity | Steps | Physical activity  Standard 1: 1.1  Standard 3: Parent/caregiver involvement in child physical activity |
| Szpunar, 2021 | Canada | Centre-based | 9 services (222 children) | Physical activity | MVPA, total physical activity, sedentary behaviour | Physical activity  Standard 1: 1.1; 1.2; 1.3; 1.4; 1.5; 1.6; 1.7; 5.1; 6.1  Standard 2: 2.4; 3.4; |
| Telford, 2021 | Australia | Centre-based | 15 services (314 children) | Physical activity | MVPA, total physical activity | Physical activity  Standard 1: 1.1; 1.4; 1.5; 1.7  Standard 2: 3.4; 4.2 |
| Trost, 2008 | USA | Centre-based | 1 service (42 children) | Physical activity | MVPA | Physical activity  Standard 1: 1.1; 1.4; 4.4  Standard 2: 3.3; 3.4 |
| Tucker, 2017 | Canada | Centre-based | 22 services (338 children) | Physical activity | MVPA, total physical activity, sedentary behaviour | Physical activity  Standard 1: 1.5; 1.6  Standard 2: 3.3; 3.4; 8.2; 8.6; Restructuring the scheduling of play opportunities to promote active play |
| Vaughn, 2021 | USA | Centre-based | 92 services (853 children) | Healthy eating  Physical activity | Diet quality  Total physical activity | Healthy eating  Standard 1: 3.1; 4.1  Standard 2: 6.7  Standard 3: 7.2; 7.5; 7.6  Physical activity  Standard 1: 1.1  Standard 2: 3.4; 4.1; 8.2; 8.3  Standard 3: Parent/caregiver involvement in child physical activity |
| Vereecken, 2009 | Belgium | Centre-based + home | 16 services (1432 children) | Healthy eating | Fruit consumption, vegetable consumption, non-core food consumption | Healthy eating  Standard 1: 3.1  Standard 2: 1.2; 1.3; 4.3; 5.6; 6.6  Standard 3: 7.2; 7.5; 7.6 |
| Wadsworth, 2020 | USA | Centre-based | 1 service (98 children) | Physical activity | MVPA | Physical activity  Standard 1:1.1; 1.7 |
| Ward, 2020 | USA | Family day care | 166 services (496 children) | Healthy eating | Diet quality | Healthy eating  Standard 2: 2; 4.3 |
| Witt, 2012 | USA | Centre-based | 17 services (263 children) | Healthy eating | Fruit consumption, vegetable consumption | Healthy eating  Standard 1: 3.1; 5.4  Standard 2: 5.7  Standard 3: 7.2; 7.5; 7.6 |
| Wolfenden, 2019 | Australia | Centre-based | 6 services (350 children) | Physical activity | MVPA, total physical activity, sedentary behaviour, counts | Physical activity  Standard 1: 1.5; 1.6  Standard 2: 3.4; 8.6; Restructuring the scheduling of play opportunities to promote active play |
| Yoong, 2020 | Australia | Centre-based | 35 services (522 children) | Healthy eating | Diet quality, fruit consumption, vegetable consumption, non-core food consumption | Healthy eating  Standard 2: 1.1; 1.3; 4.3 |

USA: United States of America; MVPA: moderate-to-vigorous physical activity

^a^ WHO standards: Standard 1 – “build children’s knowledge and skills”; Standard 2 – “provide supportive environments”; Standard 3 – “work with families/primary caregivers about healthy eating and movement behaviours” and Standard 4 – “ensure safety”

^b^ Healthy eating practice recommendations: 1.1. Nutrition standards, recommended serving sizes for foods, snacks, meals and beverages provided are aligned with national nutrition guidelines; 1.2. Provide a variety of healthy foods from the main food groups in age-appropriate portion sizes; 1.3. Specific standards for ECEC food service menus; 1.4. Water should be widely available to children at all times; 1.5. Limit serves and types of sugar-sweetened beverages; 1.6. Offer age-appropriate milk and beverages; 1.7. Keep high energy, low nutrient foods out of the childcare; 2. Develop and adopt a healthy eating policy; 2.1. Nutrition policy is reviewed; 2.2. Parents are involved in the development of the policy; 2.3. Ensure staff are willing to working within the policy; 2.4. Provide a copy of the policy to parents and staff; 3.1. Offer a variety of food awareness/education activities; 4.1 Educators are trained in nutrition curriculum/education; 4.2. Educators are trained to understand children can be healthy at a variety of weights and avoid referring to child’s body size; 4.3. Educators are trained in implementing healthy eating practices; 4.4. Food Service Staff are trained food hygiene and safety; 5.1. Food is not to be used as reward or punishment; 5.2. Avoid celebrating special occasions with food or using as a reward; 5.3. Don’t force or bribe children to eat; 5.4 Encourage children to taste different fruit and vegetables each day; 5.5. Educators should involve children in preparing food and laying and clearing the table; 5.6 Staff sit with children during meals, and role model healthy behaviors; 5.7. Educators discuss the food served with children; 6.1. Allow children adequate time to eat; 6.2 Provide healthy options in appropriate serves, and allow children to self-serve; 6.3 Continue to offer children healthy options; 6.4 Create a relaxed, enjoyable and social meal time environment; 6.5 Ensure regular and consistent meal and snack patterns; 6.6. Make fruit and vegetables snacks widely available and easily accessible; 6.7 Display healthy eating materials on the walls of the eating room; 7.1. Encourage parents to pack healthy food from home and ensure foods bought from home meet nutrition written standards; 7.2 Encourage family involvement in healthy eating at the ECEC; 7.3. Provide a copy of written nutrition guidelines to parents; 7.4 Provide parents a copy of ECEC menu; 7.5. Offer parent nutrition education as part of the ECEC program; 7.6. Make parents aware of nutrition learning activities provided to children

Physical activity practice recommendations: 1.1 Ensure physical activity is incorporated into daily routines and formal childcare curriculum; 1.2 Promote at least 180 minutes of physical activity of any intensity, spread throughout the day; 1.3 For children 3-4 years, promote at least 60 minutes of MVPA during the day; 1.4 Include opportunities for adult-led, structured physical activity; 1.5 Include opportunities for unstructured physical activity, free play; 1.6 Provide daily opportunities for activity through outdoor playtime; 1.7 Provide opportunities for children to develop and practice gross motor and movement skills; 1.8. Include culturally appropriate physical activities; 2.1. Engage staff and parent support for physical activity standards; 2.2. Seek consultation from experts annually on the physical activity programs delivered in the childcare; 2.3. Provide parent education at least 2 times a year; 2.4 Develop a written policy promoting physical activity and the removal of barriers to physical activity participation; 3.1 Staff should be trained to provide guidance to parents to encourage physical activity; 3.2. Staff should be trained to provide guidance to parents in appropriate sleep duration; 3.3. Staff should be trained in encouraging child physical activity and decreasing sedentary behavior; 3.4 Providing educator training on physical activity; 4.1 Educators should model physical activity by participating in activities; 4.2 Engage children in physical activity they enjoy, including games and sport; 4.3. Expressive play is encouraged; 4.4. Educators embed physical activity into educational activities; 4.5. Avoid punishing children for being physical active; 4.6 Avoid withholding physical activity as a punishment; 4.7. Elimination games should be avoided as well as competitive activates and games; 4.8. Engage equal participation from boys and girls in physical activity; 4.9. Celebrate special occasions with physical activity; 5.1 Children should not be sitting for extended periods for more than 30–60 min at a time; 5.2 When sedentary, children should be engaged in educational and creative pursuits, and be engaged socially; 5.3 Engage children that tend to be sedentary in active play; 6.1 No screen time is recommended for children <2 years; 6.2 No more than 1 hour of screen time/week is recommended for children aged 2 or above; 6.3. Screens should not be used/available during mealtimes or nap times; 6.4. Limit the use of screen time for educational activities or active movement programs; 6.5. Parent permission should be requested for children to participate in any screen-based activity; 6.6. Screen time should be supervised by an adult; 6.7. When offered, screen/digital media should be free from advertising, violence or should that tempt children to overuse; 6.8. Work with parents to limit overall screen time; 7.1 Include a nap within the daily routine, with regular sleep and wake-up times; 7.2. Provide an environment that provides restful sleep; 7.3. Maintain a calm nap-time routine; 8.1 Provide play equipment that encourages physical activity; 8.2 Provide simple play equipment to encourage creative play and exploration and portable play equipment that encourages indoor and outdoor play; 8.3 Provide adequate space for children to be physically active; 8.4. Ensure the outdoor area offers variety in terms of secure equipment in shade, open grass and varying surfaces; 8.5. Ensure that the educator to child ratio is fairly low;

Additional practices: Parent involvement in child physical activity; Restructuring the scheduling of play opportunities to promote active play

**Supplementary Table 2**. Assessment of healthy eating practice elements and mapped to WHO Standards for Healthy Eating, Physical Activity, Sedentary Behaviour and Sleep in ECEC Settings

| **Practice element** | **Dietary outcomes (number of studies including practice element reporting positive findings on outcome/total studies including practice element reporting on outcome=% overall)** | | | | | | **Overall assessment of practice element^a^** |
| --- | --- | --- | --- | --- | --- | --- | --- |
|  | **Diet quality** | **Fruit consumption** | **Vegetable consumption** | **Fruit and vegetable consumption** | **Unhealthy food consumption** | **Sugar-sweetened beverage consumption** |  |
| **Standard 1: Build children’s knowledge and skills** | | | | | | | |
| 3.1. Offer a variety of food awareness/education activities | 1/2=50  ± | 10/14=71  + | 12/16=75  ++ | 4/5=80  ++ | 5/6=83  ++ | 4/6=67  + | Likely beneficial |
| 4.1 Educators are trained in nutrition curriculum/education | 2/3=67 + | 7/11=64  + | 7/10=70  + | 3/4=75  ++ | 4/6=67  + | 2/2=100  ± | Likely beneficial |
| 5.4 Encourage children to taste different fruit and vegetables each day | NA | 2/3=67  + | 3/3=100  ++ | 1/1=100  ± | NA | 1/1=100  ± | Likely beneficial |
| 6.5 Ensure regular and consistent meal and snack patterns | NA | 0/1=0  ± | NA | NA | NA | NA | No conclusions possible due to lack of evidence |
| **Standard 2: Provide supportive environments** | | | | | | | |
| 1.1. Nutrition standards, recommended serving sizes for foods, snacks, meals and beverages provided are aligned with national nutrition guidelines | 2/2=100  ± | 3/3=100  ++ | 2/3=67  + | NA | 2/3=67  + | 1/1=100  ± | Likely beneficial |
| 1.2. Provide a variety of healthy foods from the main food groups in age-appropriate portion sizes | NA | 2/3=67  + | 5/5=100  ++ | NA | 2/2=100  ± | NA | Likely beneficial |
| 1.3. Specific standards for ECEC food service menus | 2/2=100  ± | 4/5=80  ++ | 5/5=100  ++ | NA | 4/4=100  ++ | NA | Likely beneficial |
| 1.4. Water should be widely available to children at all times | NA | 1/2=50  ± | 1/1=100  ± | 1/1=100  ± | 1/1=100  ± | 1/1=100  ± | No conclusions possible due to lack of evidence |
| 1.5. Limit serves and types of sugar-sweetened beverages | NA | NA | NA | NA | NA | NA | No conclusions possible due to lack of evidence |
| 1.6. Offer age-appropriate milk and beverages | NA | NA | NA | NA | NA | NA | No conclusions possible due to lack of evidence |
| 1.7. Keep high energy, low nutrient foods out of the childcare | NA | NA | NA | NA | NA | NA | No conclusions possible due to lack of evidence |
| 2. Develop and adopt a healthy eating policy^b^ | 2/2=100  ± | 4/5=80  ++ | 3/5=60  + | NA | 3/5=60  + | NA | Likely beneficial |
| 2.1. Nutrition policy is reviewed | 1/1=100  ± | 2/3=67  + | 3/3=100  ++ | NA | 3/3=100  ++ | NA | Likely beneficial |
| 2.3. Ensure staff are willing to working within the policy | 1/1=100  ± | 3/4=75  ++ | 3/4=75  ++ | NA | 2/4=50  - | 1/1=100  ± | Likely beneficial |
| 4.2. Educators are trained to understand children can be healthy at a variety of weights and avoid referring to child’s body size | NA | NA | NA | NA | NA | NA | No conclusions possible due to lack of evidence |
| 4.3. Educators are trained in implementing healthy eating practices | 4/4=100  ++ | 5/7=71  + | 6/8=75  ++ | 2/2 = 100  ± | 3/5=60  + | 1/1=100  ± | Likely beneficial |
| 5.1. Food is not to be used as reward or punishment | NA | NA | NA | NA | NA | NA | No conclusions possible due to lack of evidence |
| 5.2. Avoid celebrating special occasions with food or using as a reward | NA | NA | NA | NA | NA | NA | No conclusions possible due to lack of evidence |
| 5.3. Don’t force or bribe children to eat | NA | NA | NA | NA | NA | NA | No conclusions possible due to lack of evidence |
| 5.5. Educators should involve children in preparing food and laying and clearing the table | NA | NA | NA | NA | NA | NA | No conclusions possible due to lack of evidence |
| 5.6 Staff sit with children during meals, and role model healthy behaviors | NA | 2/5=40  - | 5/6=83  ++ | NA | 2/3=67  + | NA | Likely beneficial |
| 5.7. Educators discuss the food served with children | NA | 2/3=67  + | 3/4=75  ++ | 1/1=100  ± | 0/1=0  ± | 1/1=100  ± | Likely beneficial |
| 6.1. Allow children adequate time to eat | NA | NA | NA | NA | NA | NA | No conclusions possible due to lack of evidence |
| 6.2 Provide healthy options in appropriate serves, and allow children to self-serve | NA | NA | NA | NA | NA | 1/1=100  ± | No conclusions possible due to lack of evidence |
| 6.3 Continue to offer children healthy options | NA | 1/1=100  ± | 2/2=100  ± | 1/1=100  ± | NA | 1/1=100  ± | No conclusions possible due to lack of evidence |
| 6.4 Create a relaxed, enjoyable and social meal time environment | NA | NA | 1/1=100  ± | 1/1=100  ± | NA | 1/1=100  ± | No conclusions possible due to lack of evidence |
| 6.6. Make fruit and vegetables snacks widely available and easily accessible | NA | 1/3=33  - | 2/2=100  ± | 1/1=100  ± | 1/1=100  ± | NA | Possibly not beneficial |
| 6.7 Display healthy eating materials on the walls of the eating room | 0/1=0  ± | 0/1=0  ± | 2/2=100  ± | 1/1=100  ± | NA | NA | No conclusions possible due to lack of evidence |
| **Standard 3: Work with families/primary caregivers about healthy eating and movement behaviours** | | | | | | | |
| 2.2. Parents are involved in the development of the policy | NA | NA | NA | NA | NA | NA | No conclusions possible due to lack of evidence |
| 2.4. Provide a copy of the policy to parents and staff | NA | 3/3=100  ++ | 1/3=33  - | NA | 1/3=33  - | 1/1=100  ± | Likely beneficial |
| 7.1. Encourage parents to pack healthy food from home and ensure foods bought from home meet nutrition written standards | NA | 2/3=67  + | 2/3=67  + | NA | 0/2=0  ± | NA | Possibly beneficial |
| 7.2 Encourage family involvement in healthy eating at the ECEC | 0/1=0  ± | 3/5=60  + | 5/7=71  + | 2/3=67  + | 1/1=100  ± | 4/4=100  ++ | Likely beneficial |
| 7.3. Provide a copy of written nutrition guidelines to parents | NA | NA | 1/1=100  ± | NA | NA | NA | No conclusions possible due to lack of evidence |
| 7.4 Provide parents a copy of ECEC menu | NA | 1/1=100  ± | 2/2=100  ± | NA | 1/1=100  ± | NA | No conclusions possible due to lack of evidence |
| 7.5. Offer parent nutrition education as part of the ECEC program | 1/2=50  ± | 8/12=67  + | 9/13=69  + | 3/4=75  ++ | 3/5=60  + | 4/4=100  ++ | Likely beneficial |
| 7.6. Make parents aware of nutrition learning activities provided to children | 1/2=50  ± | 7/10=70  + | 7/10=70  + | NA | 3/3=100  ++ | 3/3=100  ++ | Likely beneficial |
| **Standard 4: Ensure children’s safety** | | | | | | | |
| 4.4. Food service staff are trained food hygiene and safety | NA | NA | NA | NA | NA | NA | No conclusions possible due to lack of evidence |

NA: Not applicable; ECEC: early childhood education and care

^a^ According to our categorisation framework

^b^ Mapped to broad practice theme

Legend:

++ Likely beneficial: ≥75% of primary studies were demonstrated positive findings (regardless of significance) on the examined outcome, where three or more studies were available

+ Possibly beneficial (more evidence needed): 51-74% of included primary studies demonstrated positive findings (regardless of significance) on the examined outcome, where three or more studies were available

- Probably not beneficial (more evidence needed): the majority (≥50%) of included primary studies demonstrated negative findings (regardless of significance) on the examined outcome, where three or more studies were available

-- Not beneficial: all included primary studies demonstrated negative findings (regardless of significance) on the examined outcome, where three or more studies were available

± No conclusions possible due to lack of evidence: Two or less primary studies examining this

NA: no primary studies included examining this.

**Supplementary Table 3.** Assessment of physical activity practice elements and mapped to WHO Standards for Healthy Eating, Physical Activity, Sedentary Behaviour and Sleep in ECEC Settings

| **Practice element** | **Physical activity outcomes (number of studies reporting positive findings/ total studies=% overall)** | | | | | **Overall assessment of practice element^a^** |
| --- | --- | --- | --- | --- | --- | --- |
|  | **MVPA** | **Total physical activity** | **Sedentary behavior** | **Counts** | **Steps** |  |
| **Standard 1: Build children’s knowledge and skills** | | | | | | |
| 1.1 Ensure physical activity is incorporated into daily routines and formal childcare curriculum | 24/34=71  + | 15/21=71  + | 17/28=61  + | 7/12=58  + | 8/11=73  + | Possibly beneficial |
| 1.2 Promote at least 180 minutes of physical activity of any intensity, spread throughout the day | 6/8=75 ++ | 4/5=80  ++ | 6/7=86  ++ | 0/1=0 ± | 0/1=0 ± | Likely beneficial |
| 1.3 For children 3-4 years, promote at least 60 minutes of MVPA during the day^b^ | 7/11=64 + | 3/3=100  ++ | 8/11=73  ++ | 0/1=0 ± | 0/2=0 ± | Likely beneficial |
| 1.4 Include opportunities for adult-led, structured physical activity | 18/27=67  + | 9/13=69  + | 15/23=65  + | 5/8=63  + | 5/8=63  + | Possibly beneficial |
| 1.5 Include opportunities for unstructured physical activity, free play | 9/14=64  + | 9/11=82  ++ | 7/11=64  + | 4/7=57  + | 3/5=60  + | Likely beneficial |
| 1.6 Provide daily opportunities for activity through outdoor playtime | 7/12=58  + | 10/11=91  ++ | 8/10=80  ++ | 3/4=75  ++ | 1/2=50  ± | Likely beneficial |
| 1.7 Provide opportunities for children to develop and practice gross motor and movement skills | 13/22=59  + | 8/13=62  + | 9/17=53  + | 1/5=20  - | 4/5=80  ++ | Likely beneficial |
| 2.2. Seek consultation from experts annually on the physical activity programs delivered in the childcare | 5/6=83  ++ | 3/3=100  ++ | 4/5=80  ++ | 1/1=100  ± | 2/2=100  ± | Likely beneficial |
| 4.4. Educators embed physical activity into educational activities | 3/5=60  + | 2/4=50  - | 2/5=40  - | 1/2=50  ± | 3/5=60  + | Possibly beneficial |
| 5.1 Children should not be sitting for extended periods for more than 30–60 min at a time | 4/7=57  + | 6/7=86  ++ | 5/9=56  + | 1/1=100  ± | 3/4=75  ++ | Likely beneficial |
| 6.1 No screen time is recommended for children <2 years | 1/1=100  ± | 1/1=100  ± | 1/1=100  ± | NA | NA | No conclusions possible due to lack of evidence |
| 6.2 No more than 1 hour of screen time/week is recommended for children aged 2 or above | 1/2=50  ± | 1/1=100  ± | 1/3=33  - | 1/2=50  ± | 1/1=100  ± | Possibly not beneficial |
| 6.3. Screens should not be used/available during mealtimes or nap times | NA | NA | NA | NA | NA | No conclusions possible due to lack of evidence |
| 6.4. Limit the use of screen time for educational activities or active movement programs | NA | NA | NA | NA | NA | No conclusions possible due to lack of evidence |
| 7.1 Include a nap within the daily routine, with regular sleep and wake-up times | 1/1=100  ± | NA | 1/1=100  ± | NA | NA | No conclusions possible due to lack of evidence |
| 7.3. Maintain a calm nap-time routine | NA | NA | NA | NA | NA | No conclusions possible due to lack of evidence |
| **Standard 2: Provide supportive environments** | | | | | | |
| 2.4 Develop a written policy promoting physical activity and the removal of barriers to physical activity participation | 2/3=67  + | 3/3=100  ++ | 3/4=75  ++ | 2/2=100  ± | NA | Likely beneficial |
| 3.3. Staff should be trained in encouraging child physical activity and decreasing sedentary behavior | 9/15=60  + | 8/12=67  + | 7/12=58  + | 1/3=33  - | 3/5=60  + | Possibly beneficial |
| 3.4 Providing educator training on physical activity | 22/28=79  ++ | 17/22=77  ++ | 17/24=71  + | 7/10=70  + | 7/10=70  + | Likely beneficial |
| 4.1 Educators should model physical activity by participating in activities | 4/5=80  ++ | 3/4=75  ++ | 3/5=60  + | 0/1=0  ± | 1/3=33  - | Likely beneficial |
| 4.2 Engage children in physical activity they enjoy, including games and sport | 12/16=75  ++ | 6/7=86  ++ | 8/11=73  + | 1/4=25  - | 1/2=50  ± | Likely beneficial |
| 4.3. Expressive play is encouraged | 4/8=50  - | 2/4=50  - | 6/9=67  + | 0/2=0  ± | 2/4=50  - | Possibly beneficial |
| 4.5. Avoid punishing children for being physical active | 0/1=0  ± | 0/1=0  ± | 0/1=0  ± | 0/1=0  ± | 0/1=0  ± | No conclusions possible due to lack of evidence |
| 4.6 Avoid withholding physical activity as a punishment | 0/1=0  ± | 0/1=0  ± | 0/1=0  ± | 0/1=0  ± | 0/1=0  ± | No conclusions possible due to lack of evidence |
| 4.9. Celebrate special occasions with physical activity | NA | NA | NA | NA | NA | No conclusions possible due to lack of evidence |
| 5.2 When sedentary, children should be engaged in educational and creative pursuits, and be engaged socially | 1/1=100  ± | 1/1=100  ± | 1/1=100  ± | NA | NA | No conclusions possible due to lack of evidence |
| 5.3 Engage children that tend to be sedentary in active play | 1/1=100  ± | 1/1=100  ± | 1/1=100  ± | NA | NA | No conclusions possible due to lack of evidence |
| 7.2. Provide an environment that provides restful sleep | NA | NA | NA | NA | NA | No conclusions possible due to lack of evidence |
| 8.1 Provide play equipment that encourages physical activity | 7/9=78  ++ | 6/8=75  ++ | 4/7=57  + | 1/3=33  - | 3/4=75  ++ | Likely beneficial |
| 8.2 Provide simple play equipment to encourage creative play and exploration and portable play equipment that encourages indoor and outdoor play | 4/7=57  + | 6/7=86  ++ | 3/5=60  + | 2/5=40  - | 2/3=67  + | Likely beneficial |
| 8.3 Provide adequate space for children to be physically active | 3/4=75  ++ | 6/7=86  ++ | 2/5=40  - | 0/2=0  ± | 1/2=50  ± | Likely beneficial |
| Restructuring the scheduling of play opportunities to promote active play^c^ | 2/4=50  - | 4/4=100  ++ | 3/3=100  ++ | 2/2=100  ± | NA | Likely beneficial |
| **Standard 3: Work with families/primary caregivers about healthy eating and movement behaviours** | | | | | | |
| 2.1. Engage staff and parent support for physical activity standards | NA | NA | NA | NA | NA | No conclusions possible due to lack of evidence |
| 2.3. Provide parent education at least 2 times a year | 4/4=100  ++ | 2/3=67  + | 1/2=50  ± | 2/3=67  + | NA | Likely beneficial |
| 3.1 Staff should be trained to provide guidance to parents to encourage physical activity | 3/3=100 ++ | 2/2=100  ± | 3/3=100  ++ | 1/2=50 ± | NA | Likely beneficial |
| 3.2. Staff should be trained to provide guidance to parents in appropriate sleep duration | NA | NA | NA | NA | NA | No conclusions possible due to lack of evidence |
| 6.5. Parent permission should be requested for children to participate in any screen-based activity | NA | NA | NA | NA | NA | No conclusions possible due to lack of evidence |
| 6.8. Work with parents to limit overall screen time | 3/4=75  ++ | 1/2=50  ± | 1/3=33  - | 1/3=33  - | NA | Likely beneficial |
| Parent involvement in child physical activity^c^ | 10/13=77  ++ | 7/10=70  + | 6/11=55  + | 5/8=63  + | 5/6=83  ++ | Likely beneficial |
| **Standard 4: Ensure children’s safety** | | | | | | |
| 1.8. Include culturally appropriate physical activities | 2/2=100 ± | NA | 1/1=100  ± | 2/3=67 + | NA | Possibly beneficial |
| 4.7. Elimination games should be avoided as well as competitive activates and games | NA | NA | NA | NA | NA | No conclusions possible due to lack of evidence |
| 4.8. Engage equal participation from boys and girls in physical activity | NA | NA | NA | NA | NA | No conclusions possible due to lack of evidence |
| 6.6. Screen time should be supervised by an adult | NA | NA | NA | NA | NA | No conclusions possible due to lack of evidence |
| 6.7. When offered, screen/digital media should be free from advertising, violence or should that tempt children to overuse | NA | NA | NA | NA | NA | No conclusions possible due to lack of evidence |
| 8.4. Ensure the outdoor area offers variety in terms of secure equipment in shade, open grass and varying surfaces | NA | NA | NA | NA | NA | No conclusions possible due to lack of evidence |
| 8.5. Ensure that the educator to child ratio is fairly low | NA | NA | NA | NA | NA | No conclusions possible due to lack of evidence |

MVPA: moderate-to-vigorous physical activity; NA: not applicable

^a^ According to our categorisation framework

^b^ Included interventions focus on increasing MVPA broadly and was not limited to the 60 minutes.

^c^ Recorded as an additional practice

Legend:

++ Likely beneficial: ≥75% of primary studies were demonstrated positive findings (regardless of significance) on the examined outcome, where three or more studies were available

+ Possibly beneficial (more evidence needed): 51-74% of included primary studies demonstrated positive findings (regardless of significance) on the examined outcome, where three or more studies were available

- Probably not beneficial (more evidence needed): the majority (≥50%) of included primary studies demonstrated negative findings (regardless of significance) on the examined outcome, where three or more studies were available

-- Not beneficial: all included primary studies demonstrated negative findings (regardless of significance) on the examined outcome, where three or more studies were available

± No conclusions possible due to lack of evidence: Two or less primary studies examining this

NA: no primary studies included examining this.
